# Examining the Role of *SORL1* in Driving Alzheimer's Disease‐Related Alterations in Endolysosomal Trafficking in Neurons and Microglia

**DOI:** 10.1002/alz70855_105337

**Published:** 2025-12-24

**Authors:** Brooke A. DeRosa, Yalun Zhang, Charles G. Golightly, Lauren E. Coombs, Lauren E. Rothschild, Brian W Kunkle, Michael L Cuccaro, Jeffery M Vance, Margaret Pericak‐Vance, Peter St George‐Hyslop, Derek M. Dykxhoorn

**Affiliations:** ^1^ John P. Hussman Institute for Human Genomics, University of Miami Miller School of Medicine, Miami, FL, USA; ^2^ University of Toronto, Toronto, ON, Canada; ^3^ Dr. John T. Macdonald Foundation Department of Human Genetics, University of Miami Miller School of Medicine, Miami, FL, USA; ^4^ University of Miami Miller School of Medicine, Miami, FL, USA; ^5^ Department of Neurology, University of Miami Miller School of Medicine, Miami, FL, USA

## Abstract

**Background:**

Disruption of endolysosomal trafficking is a key pathogenic driver in Alzheimer's disease (AD). Among the genes linked to the endosomal pathway in AD, *SORL1* is recognized as one of the most significant risk factors. *SORL1* encodes an endocytic sorting receptor that regulates endosomal trafficking and processing of crucial AD‐related molecules, including pathogenic forms of amyloid‐β (e.g., Aβ42) and amyloid precursor protein (APP). Using complementary cell‐based models, we examined the impact of a protein‐truncating variant of *SORL1* on endolysosomal trafficking and APP processing.

**Method:**

Induced pluripotent stem cell (iPSC) lines were generated from two siblings with early‐onset AD carrying a rare protein‐truncating deletion in *SORL1* (rs1343336951; p.C1431fs). *SORL1* wild‐type isogenic control iPSC lines were created using CRISPR/Cas9 from these patient‐derived lines. Following validation, the iPSCs were differentiated into forebrain neurons and microglia to assess the impact of the *SORL1* deletion on endolysosomal trafficking in each cell‐type. Additional analyses were performed in HEK293‐APPswe cells overexpressing either wild‐type *SORL1* or the C1431fs variant.

**Result:**

In HEK293‐APPswe cells, the C1431fs variant enhanced secretion of Aβ42 (*p* <0.01), Aβ40 (*p* <0.01), as well as soluble α‐secretase (sAPPα; *p* <0.01) and β‐secretase (sAPPβ; *p* <0.01) cleavage products. Furthermore, C1431fs led to an increase in the secretion of soluble SORL1 (*p* <0.01). Surface biotinylation experiments showed that C1431fs reduced levels of SORL1 at the cell surface (*p* <0.05), while increasing the surface levels of APP (*p* <0.05). In *SORL1* ^+/C1431fs^ neurons, we observe an accumulation of APP in early endosomes (*p* = 0.002), endosomal swelling (*p* = 0.004), and a higher number of early endosomes per cell (*p* = 0.018). Additionally, *SORL1* ^+/C1431fs^ neurons trend toward increased secretion of Aβ42 compared to controls. Our analysis of phagocytosis in *SORL1* ^+/C1431fs^ microglia, assessed by the uptake of pHrodo‐labeled fibrillar Aβ42, suggests that the deletion reduces phagocytic activity. Current investigations in microglia are evaluating the concentration of specific cytokines and chemokines secreted in response to proinflammatory stimuli.

**Conclusion:**

Our findings suggest that the *SORL1* C1431fs deletion is capable of inducing defects in endolysosomal trafficking in both neurons and microglia. Ongoing studies in *SORL1* ^+/C1431fs^ microglia and neurons will further expand our understanding of SORL1's role in regulating endolysosomal phenotypes across multiple cell types implicated in AD.

